# Supplementary material for: MetaQTL: a package of new computational methods for the meta-analysis of QTL mapping experiments
Source: BMC Bioinformatics. 2007 Feb 8;8:49. doi: 10.1186/1471-2105-8-49 (PMC1808479; doi:10.1186/1471-2105-8-49)
Supplement: Additional File 7 — MetaQTL Package : jar file and tutorial. This Zip archive contains both the MetaQTL JAR file and the files of the tutorial. [file 1471-2105-8-49-S7.zip › org.inra.metaqtl/doc/org/metaqtl/bio/entity/package-use.html]

Uses of Package org.metaqtl.bio.entity


|  |  |  |
| --- | --- | --- |
| **Overview** \| **Package** \| Class \| **Use** \| **Tree** \| **Deprecated** \| **Index** \| **Help** | |  |
| PREV   NEXT | **FRAMES**    **NO FRAMES**     **All Classes** |  |


---


## **Uses of Package org.metaqtl.bio.entity**

| Packages that use org.metaqtl.bio.entity | |
| --- | --- |
| **org.metaqtl.bio.entity** |  |
| **org.metaqtl.util** |  |

Classes in org.metaqtl.bio.entity used by org.metaqtl.bio.entity

****AlleleContainer****
  
          Class Description Here

****BioEntity****
  
          Class Description Here

****BioEntityContainer****
  
          Class Description Here

****GeneticMap****
  
          Class Description Here

****GroupContainer****
  
          Class Description Here

****IndividualContainer****
  
          Class Description Here

****Locus****

****LocusContainer****
  
          Class Description Here

****OntologyTerm****

****Population****
  
          Class Description Here

Classes in org.metaqtl.bio.entity used by org.metaqtl.util

****CrossPopulation****
  
          Class Description Here

---


|  |  |  |
| --- | --- | --- |
| **Overview** \| **Package** \| Class \| **Use** \| **Tree** \| **Deprecated** \| **Index** \| **Help** | |  |
| PREV   NEXT | **FRAMES**    **NO FRAMES**     **All Classes** |  |


---
